# Supplementary figures and images for: Pangenomic antiviral effect of REP 2139 in CRISPR/Cas9 engineered cell lines expressing hepatitis B virus surface antigen
Source: PLoS One. 2023 Nov 1;18(11):e0293167. doi: 10.1371/journal.pone.0293167 (PMC10619774; doi:10.1371/journal.pone.0293167)

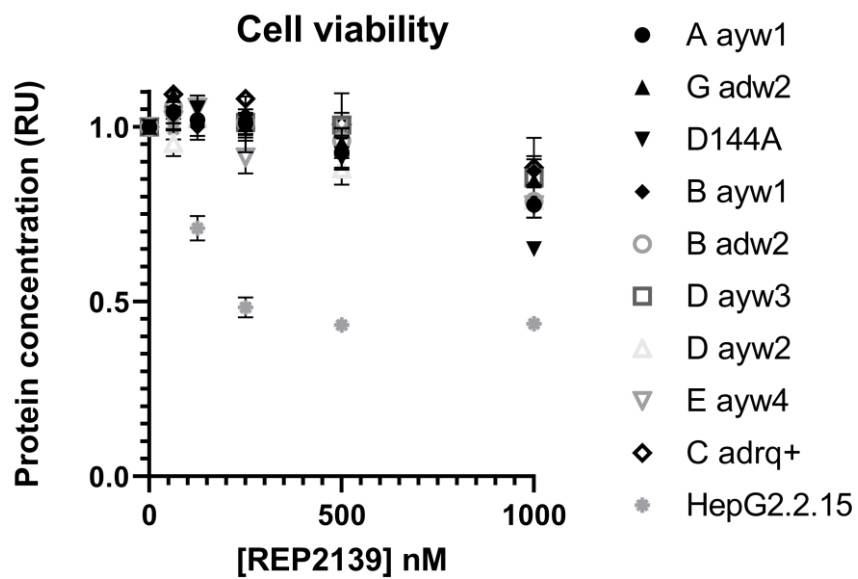

**S1 Fig**

Supplement: S1 Fig — Cell viability was assessed for the indicated cell lines for each REP 2139 concentration, by performing a BCA protein assay and measurement of the absorbance at 560nm. RU, relative unit. (PDF) [file pone.0293167.s001.pdf]
