# Supplementary material for: Pangenomic antiviral effect of REP 2139 in CRISPR/Cas9 engineered cell lines expressing hepatitis B virus surface antigen
Source: PLoS One. 2023 Nov 1;18(11):e0293167. doi: 10.1371/journal.pone.0293167 (PMC10619774; doi:10.1371/journal.pone.0293167)
Supplement: S1 Table — One-way ANOVA analysis followed by a Dunnet’s comparison between various REP 2139 concentration and 0nM REP 2139 were performed. (*, P < 0.05; **, P < 0.01; ***, P < 0.001; ****, P < 0.0001). Statistical analyses were performed using Prism-GraphPad. (PDF) [file pone.0293167.s002.pdf]

S1 Table. Statistical analyses results of REP 2139 antiviral effect. One-way ANOVA analysis followed by a Dunnet's comparison between various REP 2139 concentration and 0nM REP 2139 were performed. (*, $P < 0.05$; **, $P < 0.01$; ***, $P < 0.001$; ****, $P < 0.0001$). Statistical analyses were performed using Prism-GraphPad.

Dunnet's test		Cell lines									
		A ayw1	B ayw1	B adw2	C adrq+	D ayw2	D ayw3	E ayw4	G adw2	D144A	HepG2.2.15
Comparison between [REP 2139] nM	0 vs 62,5	**	ns	*	*	ns	ns	ns	*	*	****
	0 vs 125	***	ns	*	***	ns	ns	*	**	*	****
	0 vs 250	****	**	**	***	ns	ns	**	***	**	****
	0 vs 500	****	****	***	****	*	ns	****	****	***	****
	0 vs 1000	****	****	****	****	**	*	***	****	****	****
