# Supplementary material for: Pangenomic antiviral effect of REP 2139 in CRISPR/Cas9 engineered cell lines expressing hepatitis B virus surface antigen
Source: PLoS One. 2023 Nov 1;18(11):e0293167. doi: 10.1371/journal.pone.0293167 (PMC10619774; doi:10.1371/journal.pone.0293167)
Supplement: S1 Raw images — (PDF) [file pone.0293167.s003.pdf]

Agarose gel (1%) :

Molecular weight :
Thermofisher 1 Kb Plus
DNA Ladder
(10787018)

Captured with Azure
Biosystems. UV 302nm

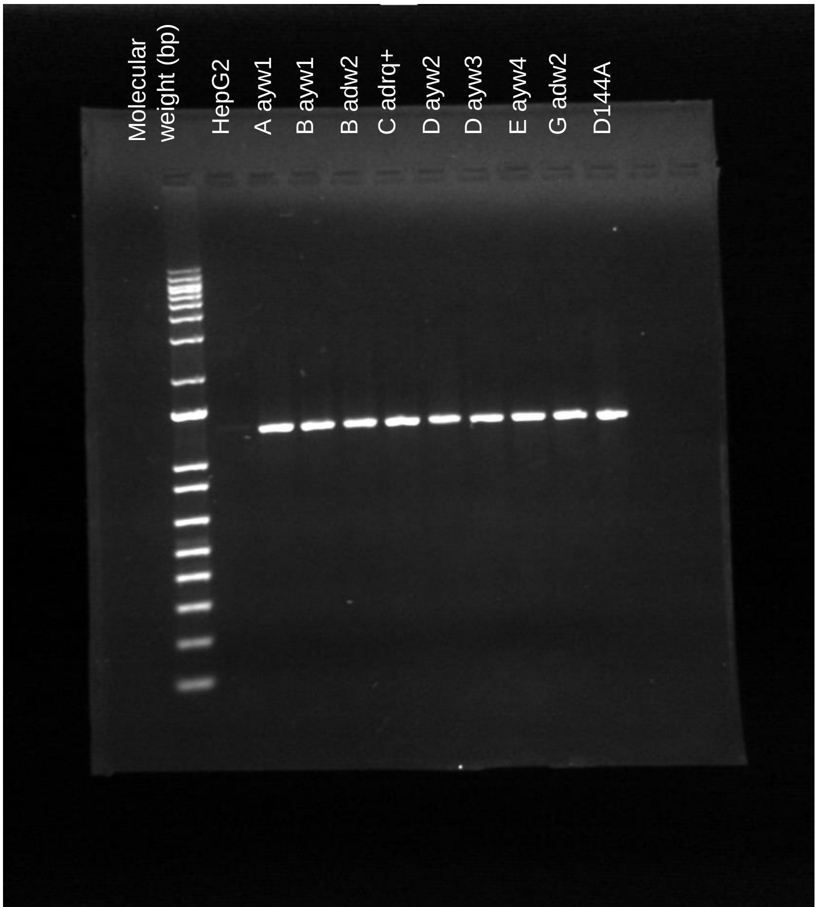

Figure 3B

Agarose gel (1%) :

Molecular weight :
Thermofisher 1 Kb Plus
DNA Ladder
(10787018)

Captured with Azure
Biosystems. UV 302nm

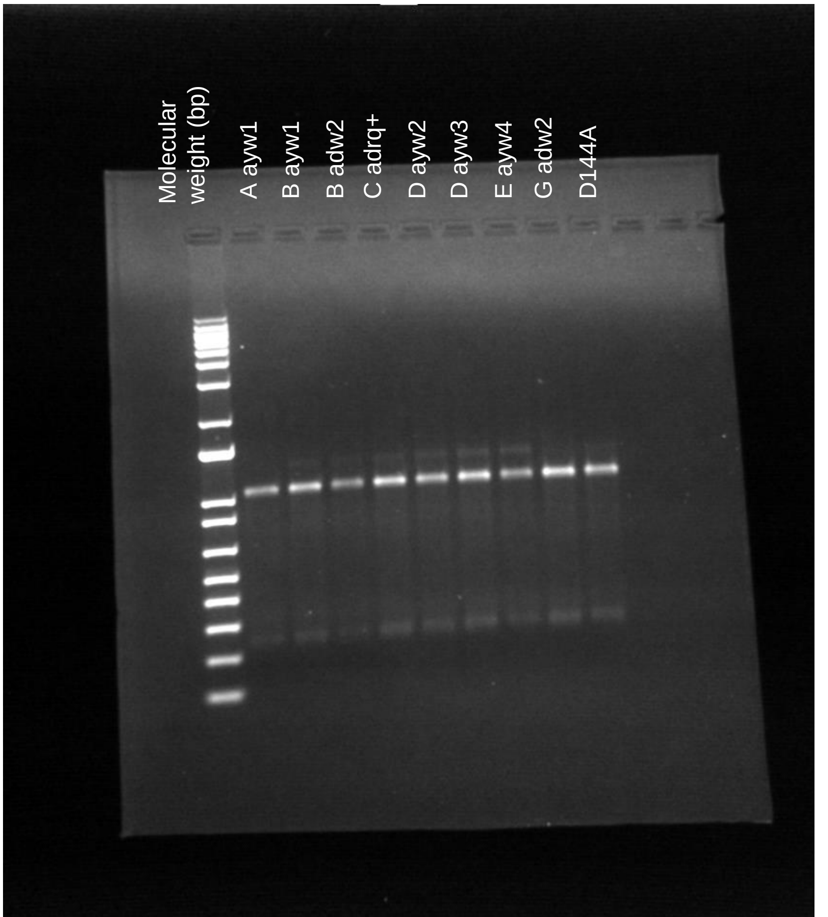

Figure 3C
